# Inorganic Solar Cells Based on Electrospun ZnO Nanofibrous Networks and Electrodeposited Cu_2_O

**DOI:** 10.1186/s11671-015-1169-8

**Published:** 2015-12-01

**Authors:** Luming Zhang, Huaquan Sun, Lai Xie, Jinnan Lu, Luyong Zhang, Sujuan Wu, Xingsen Gao, Xubing Lu, Jinhua Li, Jun-Ming Liu

**Affiliations:** Institute for Advanced Materials and Guangdong Provincial Key Laboratory of Quantum Engineering and Quantum Materials, South China Normal University, Guangzhou, 510006 China; Faculty of Materials Science and Engineering, Hubei University, Wuhan, 430062 China; Laboratory of Solid State Microstructures, Nanjing University, Nanjing, 210093 China

**Keywords:** Electrospun ZnO nanofibers, Electrodeposited Cu_2_O layer, pH value, Electrodeposition time, ZnO/Cu_2_O solar cells

## Abstract

The nanostructured ZnO/copper oxide (Cu_2_O) photovoltaic devices based on electrospun ZnO nanofibrous network and electrodeposited Cu_2_O layer have been fabricated. The effects of the pH value of electrodeposition solution and the Cu_2_O layer thickness on the photovoltaic performances have been investigated. It is revealed that the pH value influences the morphology and structure of the Cu_2_O layer and thus the device performances. The Cu_2_O layer with an appropriate thickness benefits to charge transfer and light absorption. The device prepared at the optimal conditions shows the lowest recombination rate and exhibits a power conversion efficiency of ~0.77 %.

**Electronic supplementary material:**

The online version of this article (doi:10.1186/s11671-015-1169-8) contains supplementary material, which is available to authorized users.

## Background

Inexpensive solar cells that can be synthesized from solutions on various low-cost substrates are of particular interest for distributed electricity generation [1–3]. A series of advantages such as material abundance, low toxicity, and high stability are identified for these “ultra-low-cost” cells [4, 5]. Obviously, all-oxide photovoltaics have these potential advantages. For example, copper oxide (Cu_2_O) has been recognized as one of the promising photovoltaic materials due to its abundance, high absorption coefficient, low-cost fabrication, and high theoretical power conversion efficiency (PCE) of ~20 % [6]. In fact, various techniques such as electrodeposition, sputtering, and thermal oxidation of metallic Cu sheet were once used for fabricating Cu_2_O films for photovoltaic devices [7–9]. Among them, electrodeposition is easily down-scaled and can produce extremely uniform films on conductive substrates, allowing an attractive potential to synthesize inexpensive Cu_2_O photovoltaics on a variety of supporting substrates with minimal energy input [10]. To date, the Cu_2_O photovoltaic layers were used in a number of heterojunction solar cells [11–16].

ZnO as a wide-bandgap semiconductor (approximately 3.3 eV) with high electron mobility has been found to be the most stable and efficient. Fortunately, ZnO nanostructures with different morphologies can be cheaply synthesized by solution method [17–19]. A combination of Cu_2_O and ZnO to fabricate heterojunction solar cells has thus been receiving attention recently. Nevertheless, ZnO/Cu_2_O heterojunction solar cells fabricated by magnetron-sputter deposition only show a PCE of 0.24 % [8]. Solution-processed 3D ordered Al-doped ZnO (AZO)-ZnO/Cu_2_O solar cells based on patterned ZnO nanorod arrays and electrodeposited Cu_2_O films demonstrate a maximum PCE of 1.52 % [9]. These exciting results indicate that one can improve the performance of ZnO/Cu_2_O solar cells by taking advantage of nano-heterojunctions. A high efficiency of 6.1 % was reported for Cu_2_O-based heterojunction solar cells prepared by thermally oxidizing copper sheets [7]. With respect to the cells based on high-energy-cost copper oxidation, the electrodeposited and sputtered ZnO/Cu_2_O cells show worse performances. The important reason is the lower minority carrier transport length and shorter collection length for photo-generated charges in the Cu_2_O layer.

In order to enhance the interfacial areas in ZnO/Cu_2_O structure and reduce the minority carrier collection length, ZnO nanostructures were once extensively used [18, 20–22]. However, the efficiency of the nanostructure cells is lower than that of the best bilayer planar cells. This is due to the incompatibility between the short length required for good charge collection and the longer length for the formation of full built-in potential (*V*_bi_) for inhibition of recombination [23]. In order to balance the charge collection and conformation of *V*_bi_ length, it is necessary to explore new strategy to engineer the ZnO/Cu_2_O interface and nanostructures.

Electrospinning technique provides a simple, cost-effective, and template-free process with potentials to prepare various materials with one-dimensional (1D) nanostructures for large-scale production [22, 24]. High-surface-area 1D nanofibrous networks have the potentials to improve the performances of solar cells because of increased interface area and improved charge carrier collection. The as-prepared electrospun nanofibers were already used in various solar cells [19, 25]. To our best knowledge, implication of ZnO nanofibers (ZnO-NFs) in ZnO/Cu_2_O solar cell devices has not yet been reported so far. On the other hand, an open-circuit voltage (*V*_oc_) of 1.2 V was reported for Cu_2_O device fabricated by oxidizing Cu sheets in a controlled atmosphere [26], while the reported *V*_oc_ for electrodeposited bilayer cells only ranges from 0.19 to 0.59 V [9, 27, 28]. These results suggest that the *V*_oc_ may be sensitive to the electrodeposition processes. In order to improve the performance of cells based on ZnO nanostructure and electrodeposited Cu_2_O, the parameters in the electrodeposition process needs be optimized.

Based on these considerations, inorganic solar cells with electrodeposited Cu_2_O layer and electrospun ZnO nanofibrous networks are fabricated in this work. Here, ZnO nanofibrous networks are employed to electrodeposit Cu_2_O, so that effective radial junctions across the interfaces can be formed, benefiting to rapid charge separation and transport [21, 29]. The key issue here is to design a continuous nanofiber heterojunction with a sufficient interfacial area for the efficient charge transfer. Though the effect of pH value on the performance of ZnO/Cu_2_O solar cells has been reported previously [30, 31], the electrodeposition processes impact the electrical performance significantly. Thus, the effects of the pH value and the duration on the performances of ZnO-NFs/Cu_2_O and relative mechanism have been further investigated. Subsequently, we investigate the performances and underlying device physics of these inorganic solar cells based on the ZnO nanostructures. It is found that the recombination rate decreases dramatically at the optimized process and an efficiency of 0.77 % is demonstrated.

## Methods

### Materials and Methods

**Fig. 1:**
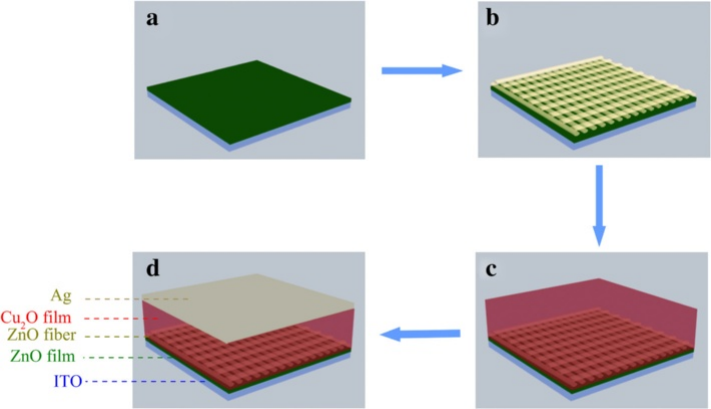
(Color online) A schematic drawing of the device based on electrospun ZnO nanofibrous networks and electrodeposited Cu_2_O. **a** ZnO film spin-coated on ITO. **b** ZnO nanofibrous networks. **c** Cu_2_O electrodeposited on ZnO nanofibrous networks. **d** Ag electrode evaporated

Figure 1 shows a schematic of the as-prepared solar cells. For each sample, cleaned indium tin oxide (ITO) substrate was first coated with a compact ZnO film of about 80 nm in thickness by spin-coating the sol-gel solution (0.5 M zinc acetic and 0.5 M mono-ethanolamine in 2-methoxyethanol) at 2000 rpm. The ZnO nanofibrous networks were then electrospun at a flow rate of 0.2 ml/h onto the surface of the ZnO film from a precursor gel containing zinc acetic dehydrate (10 %) and poly(vinylpyrrolidone) (PVP) (3 %) in the solution of isopropanol and 2-methoxyethanol (1:1 in volume) in air ambient. For this electrospinning, a voltage of 8.0 kV was applied between a metal orifice and a grounded rotating collector with a distance of 8 cm for 45 min. Subsequently, these samples were annealed at 450 °C for 30 min in oxygen by rapid thermal processing (RTP) to remove organic components and allow grain nucleation and growth of the ZnO-NFs. Here, the heating rate was 3 °C/s during the calcination. After the annealing, the samples were cooled down naturally in O_2_. Then, the samples were submitted to electrodeposit Cu_2_O layer in an aqueous solution of 0.4 M copper sulfate hydrate and 3 M lactic acid.

The pH value of electrodeposition solution was adjusted with saturated NaOH aqueous solution from 8.0 to 13.0. The deposition temperature was fixed at 60 °C. The deposition voltage was set at −0.3 V versus Ag/AgCl reference electrode. The thickness of Cu_2_O layer was controlled by the growth time, which was changed from 10 to 60 min. In order to reduce interfacial defects and improve the interfacial contact between the ZnO nanofibrous networks and Cu_2_O layer, ZnO powder was added to the buffered solution to protect the ZnO from etching during the electrodeposition [15]. After the Cu_2_O deposition, the as-prepared samples were rinsed thoroughly with deionized water and dried in air and then annealed in a glove-box filled with high-purity N_2_ at 100 °C for 120 min. Finally, an Ag electrode was evaporated on the sample surface through a shadow mask under a vacuum of 10^−4^ Pa. To this stage, the solar cells were fabricated with the standard in-plane size of 2 mm × 4 mm. The morphology, dimensions, and crystallinity were characterized by transmission electron microscopy (TEM, F2010, Japan), scanning electron microscopy (SEM, JEOL 5700, Japan), and X-ray diffraction (XRD, X’Pert PRO, Cu Ká radiation), respectively.

### Device Characterizations

The photovoltaic performances of these inorganic solar cell devices were characterized using a Keithley 2400 source meter under an illumination of 100 mW cm^−2^ (Newport 91160, 150 W solar simulator equipped with an AM1.5G filter). The radiation intensity was calibrated by a standard silicon solar cell (certified by NREL) as the reference. The external quantum efficiency (EQE) and the UV-vis absorption spectra were measured using a standard EQE system (Newport 66902). The electrochemical impedance spectroscopy (EIS) measurements in the device configuration were performed on the Zahner Zennium electrochemical workstation in the dark. A 30 mV AC sinusoidal signal source was employed over the constant bias with the frequency ranging from 1 Hz to 4 MHz. The obtained impedance data were fitted with a proper equivalent circuit by Scribner Associate Z-View software. The Mott-Schottky (M-S) measurements were carried out by a standard electrochemical measurement at a 1-kHz frequency with AC amplitude of 20 mV in a 0.1 M Na_2_SO_4_ solution in dark. A Pt foil serves as the counter electrode, and the Ag/AgCl (statured with 1 M KCl solution) electrode was used as reference electrode.

## Results and Discussion

**Fig. 2:**
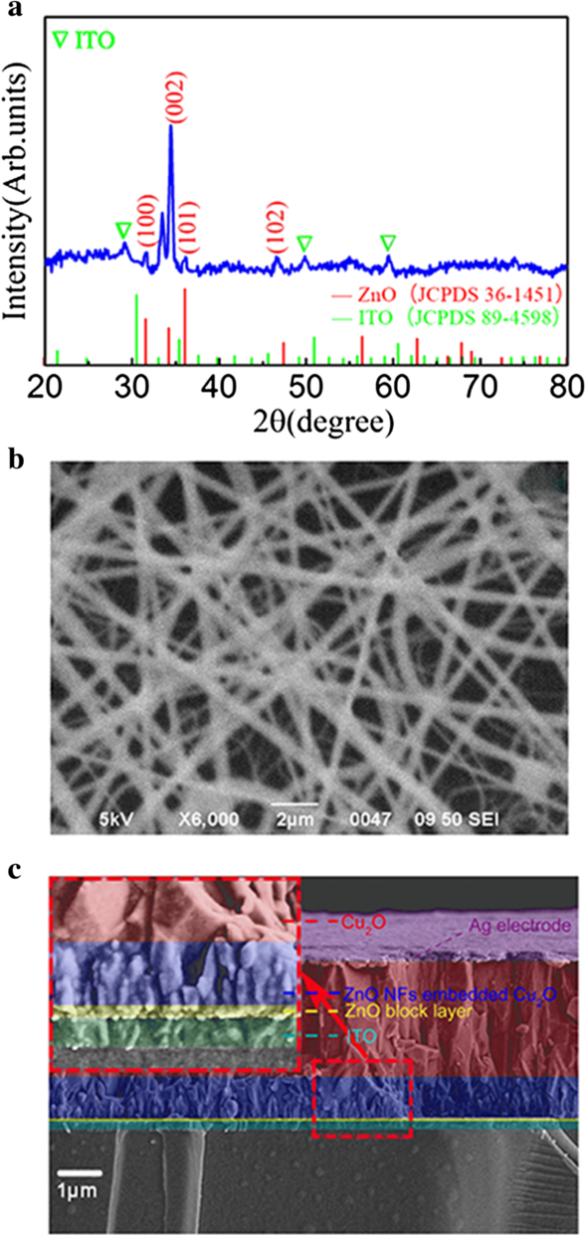
(Color online) **a** XRD spectrum of the ZnO-NFs. **b** SEM image of the ZnO-NFs. **c** cross-sectional SEM view of the ZnO-NFs/Cu_2_O solar cells

To fabricate the nanostructured photovoltaics, good space filling of Cu_2_O into the ZnO nanofibrous networks is essential to form junctions. Figure 2 shows the SEM image and XRD spectra of ZnO nanofibrous networks, as well as the cross-sectional SEM image of the ZnO/Cu_2_O device. The XRD pattern in Fig. 2a indicates that the ZnO-NFs have hexagonal wurtzite structure (JCPDS 36-1451) with the (002)-preferred orientation [32, 33]. As shown in Fig. 2b, these ZnO-NFs have a diameter distribution over 100–300 nm and they cross over one and another. Such fibers crossing can improve the effective interfacial contact between ZnO and Cu_2_O, benefiting to the charge transfer [24]. Fig. 2c exhibits the cross-sectional SEM view of the ZnO-NFs/Cu_2_O device. Clearly, the ZnO-NFs are well embedded inside the Cu_2_O layer. No obvious microscopic defects such as pores or pinholes were observed throughout the film thickness, suggesting that the microstructural quality is quite good. The thickness of the ZnO/Cu_2_O active layer is about ~3.0 μm.

**Fig. 3:**
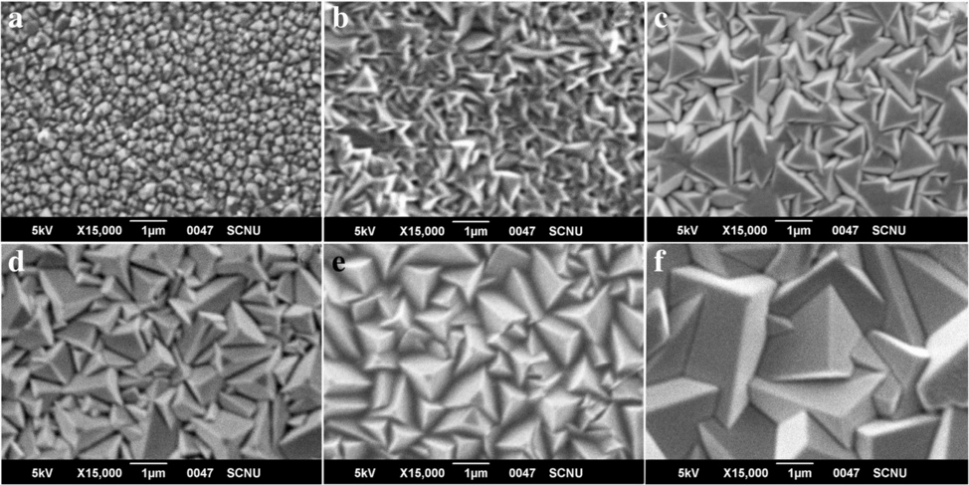
(Color online) Top view of SEM images of the electrodeposited Cu_2_O layer on ZnO nanofibrous networks at different pH values: **a** 8, **b** 9, **c** 10, **d** 11, **e** 12, and **f** 13

**Fig. 4:**
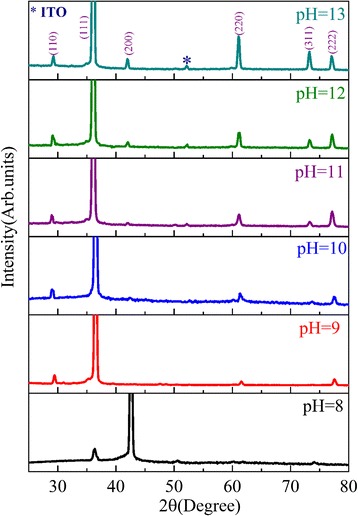
(Color online) XRD spectra for Cu_2_O thin film electrodeposited on ZnO nanofibrous networks at different pH values for 30 min and then annealed at 100 °C for 2 h in an N_2_ atmosphere. They are the characteristic XRD peaks of cubic structure (JCPDS 05-0667)

Now, one comes to look at the microstructures of the Cu_2_O layers prepared at different pH values of the electrodeposition solution, and the SEM images and XRD data are shown in Figs. 3 and 4, respectively. It is shown clearly that the morphology of Cu_2_O film depends on the pH value. The top-view SEM images indicate that the Cu_2_O film consist of agglomerates of pyramids crystallites which are dominant in the film deposited at pH = 8 in particular. The crystallites prefer the triangular shape at pH~9 or 10, and truncated pyramid shape at pH~11 to 13, similar to the textured surface microstructures of crystalline silicon solar cells [34–36]. The grain size can be very different if the pH value varies, indicating increasing grain size with increasing pH value. It is believed that less grain boundary defects will be available for the cases of larger grains, benefiting to the charge transfer and recombination reduction.

**Fig. 5:**
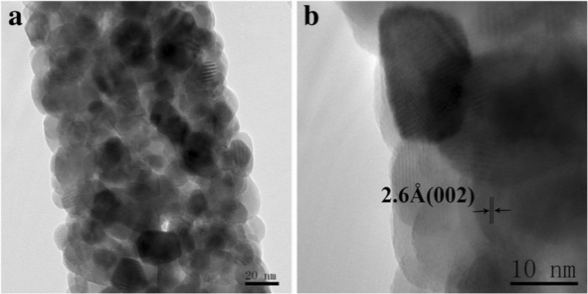
(Color online) **a** TEM image of an electrospun ZnO nanofiber. **b** HTEM image of the ZnO nanofiber

**Fig. 6:**
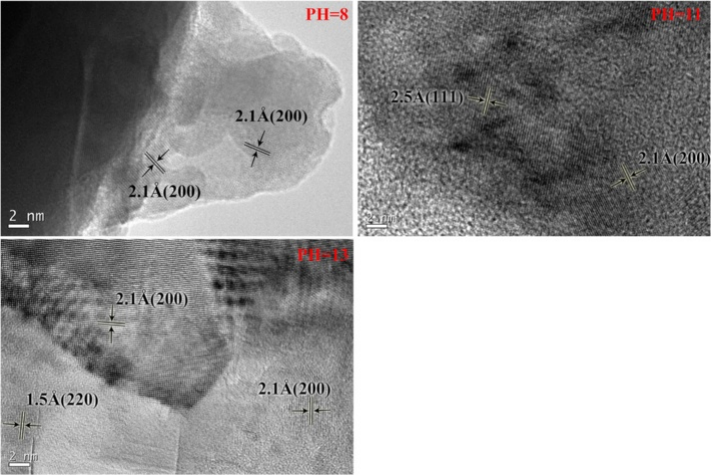
(Color online) HTEM images of Cu_2_O films electrodeposited at different pH values

Figure 4 shows the XRD patterns for a series of Cu_2_O films deposited at different pH values. The films are polycrystalline and have cubic structure (JCPDS 05-0667). The films prefer the (200)-texture at low pH values (e.g., pH = 8) and (111)-texture at high pH values (e.g., pH = 9–13), agreeing with earlier results [11, 22, 37, 38]. It is noted that the (002)-oriented ZnO-NFs favor better matching with the (111)-texture Cu_2_O film [39]. On the basis of this good crystallographic matching, formation of interface states during the heterojunction epitaxial growth can be restrained, which will effectively limit the recombination of electrons in n-ZnO with holes in p-Cu2O at the interface region and enhance *V*_bi_ [9]. At pH values higher than 11, the Cu_2_O (200) peak appears and the intensity increases with increasing pH value. The (220) and (311) reflections can also be observed at pH value higher than 9, while similar results were reported in literature [22, 40]. Figures 5 and 6 show the HTEM images of ZnO nanofiber and electrodeposited Cu_2_O, respectively. As seen in Figs. 5 and 6, the lattice fringes of ZnO nanofiber and Cu_2_O electrodeposited at pH values of 8, 11, and 13 can be seen clearly. It indicates that the crystallinity of ZnO nanofiber and Cu_2_O films electrodeposited at different pH values is good.

**Fig. 7:**
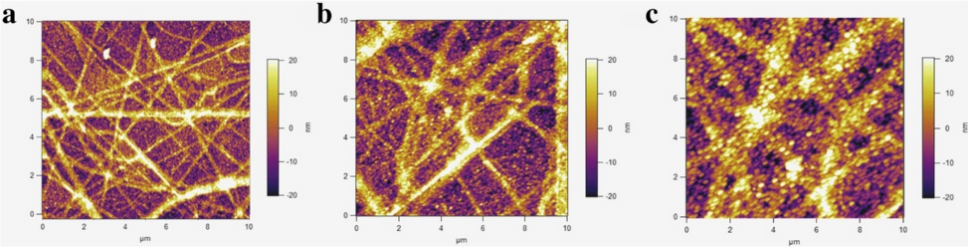
(Color online) AFM images evidencing the grain growth of the electrodeposited Cu_2_O layer on the ZnO-NFs with the deposition time of **a** 5, **b** 10, and **c** 20 s, respectively

Figure 7 shows the AFM images of the surface morphology for a series of samples upon the Cu_2_O electrodeposition for different times (growth time). One sees no identifiable Cu_2_O grain growth in the initial 5~10 s, while remarkable grain growth can be identified at ~20 s, as shown in Fig. 7c. At this stage, the spaces between neighboring ZnO-NFs are almost fulfilled with the Cu_2_O grains, suggesting that the Cu_2_O grain growth is a bottom-up process. The AFM observations (Fig. 7) are consistent with the cross-sectional SEM image. In short, it is revealed that the preferred orientations and morphologies of the as-deposited Cu_2_O films onto the ZnO-NFs, and thus the performances of the as-prepared solar cells, can be significantly controlled by the pH value of the electrodeposition solution.

**Fig. 8:**
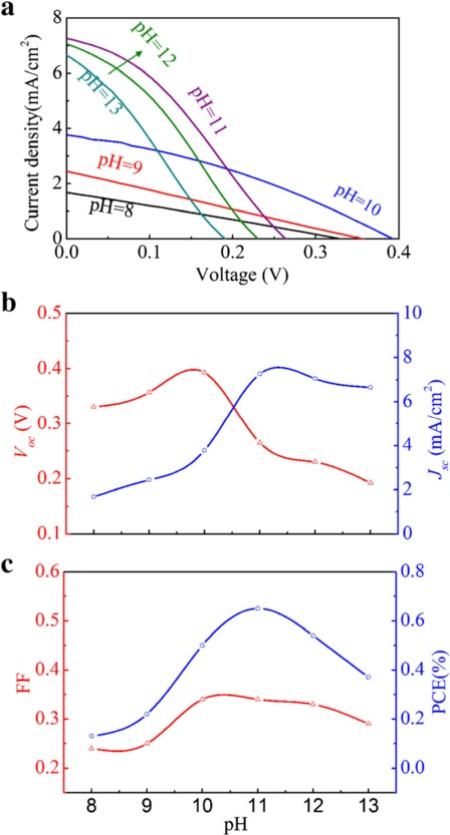
(Color online) **a** Current-voltage (*J*-*V*) curves of ZnO-NFs/Cu_2_O devices for Cu_2_O layer fabricated at different pH value. **b** Measured *V*_oc_ and *J*_sc_. **c** FF and PCE as a function of pH value for Cu_2_O layer

Along this line, it is understood that the solution pH value must be optimized in order to achieve the best cell performances. Figure 8a shows the measured current density-voltage (*J-V*) curves for a series of as-prepared solar cells where the Cu_2_O films are deposited at different pH values. Figure 8b, c shows the measured photovoltaic parameters including *V*_oc_, short-circuit current density (*J*_sc_), fill factor (FF), and PCE as functions of the pH value. It is seen that these parameters all increase with increasing pH value in the low-pH-value range. The optimized parameters—*J*_sc_~7.26 mA/cm^2^, *V*_oc_~0.265 V, FF~0.34, and PCE~0.65 %—are obtained at the pH value of 11, the optimal value. Additional file 1: Figure S1 shows the (111)/(200) peak intensity ratio as a function of pH value. It can be seen that the (111)/(200) peak intensity ratio reaches maximum at pH = 11. It is known that the performance of Cu_2_O cells is strongly related to the morphology and structural property of Cu_2_O layer [41–43]. The device with the higher ratio of (111)-preferred orientation results in better performance than that of the (200)-preferred orientation structure. This is consistent with the previous reports [41–43].

We have fabricated the cells with planar ZnO structure instead of ZnO nanofibrous networks for comparison. Additional file 1: Figure S2 shows the *J-V* curves for the reference cell and the present cell. The reference device yields a *J*_sc_ of 3.17 mA/cm^2^, a *V*_oc_ of 0.316 V, a FF of 0.328, and a PCE of 0.328 %. It can be seen that the ZnO nanofibrous networks do benefit to higher *J*_sc_ and thus higher PCE. It was reported that alkaline solution for depositing Cu_2_O does not erode the ZnO-NFs [21]. The higher-than-optimal pH value yields the lower PCE, mainly attributed to the decreases in *V*_oc_ and FF. It is known that *V*_oc_ is not only controlled by the band alignment of Cu_2_O and ZnO-NFs but also the heterojunction quality [22, 38, 44].

**Fig. 9:**
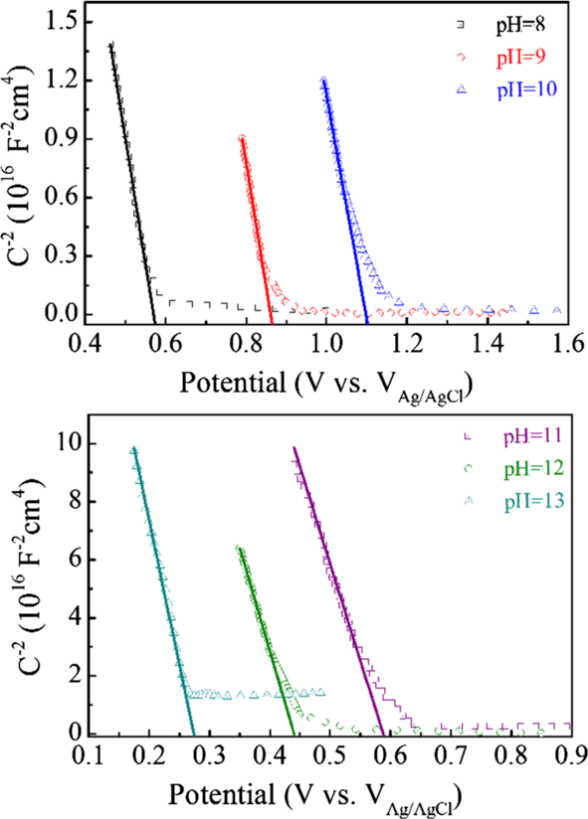
(Color online) The M-S plots for Cu_2_O layers deposited at different pH values on ITO-glass substrates

In order to understand comprehensively the reasons for the substantial impact of the pH value, we perform series electrical and electrochemical characterizations on the solar cell microstructures. Figure 9 shows the Mott-Schottky (M-S) plots for the Cu_2_O films which are directly electrodeposited on ITO-glass substrates at different pH values. The slopes of the M-S plot can be used to calculate the carrier concentrations of the Cu_2_O films [45, 46]. The hole concentration increases with increasing pH value and reaches the maximal of ~1.98 × 10^14^ cm^−3^ at the pH value of ~10. This concentration is comparable to the typical value of 10^13^–10^14^ cm^−3^ for electrodeposited Cu_2_O in earlier reports [23, 28, 47]. These concentrations are several orders of magnitude lower than the measured electron concentrations (e.g., ~7.95 × 10^18^ cm^−3^) of the ZnO-NFs, as evaluated from the M-S plots presented in Additional file 1: Figure S3. Thus, the Fermi level of the ZnO-NFs can be assumed as constant. It is known that a higher hole concentration induces a more positive Fermi level. The magnitude of the *V*_bi_ corresponds to the Fermi level difference between the Cu_2_O and ZnO-NFs [45]. It is noted that the Fermi level difference in ZnO-NFs/Cu_2_O cell with the Cu_2_O deposited at the pH value of ~10 (ZnO-NFs/Cu_2_O (pH 10)) is larger than the others. Thus, the ZnO-NFs/Cu_2_O (pH 10) device shows the largest *V*_bi_. *V*_oc_ of the device is mainly determined by the *V*_bi_ [20, 23]. This explains why the ZnO-NFs/Cu_2_O (pH 10) device has the highest *V*_oc_. Additional evidence comes from the impedance spectra for the solar cells prepared under different pH values for the Cu_2_O deposition, as given in Additional file 1: Figure S4. Although the cell at the pH value of ~11 has the largest recombination resistance, the highest *V*_oc_ appears at the pH value of ~10. Since the *V*_oc_ of a solar cell is mainly determined by its *V*_bi_ [20, 23].

**Fig. 10:**
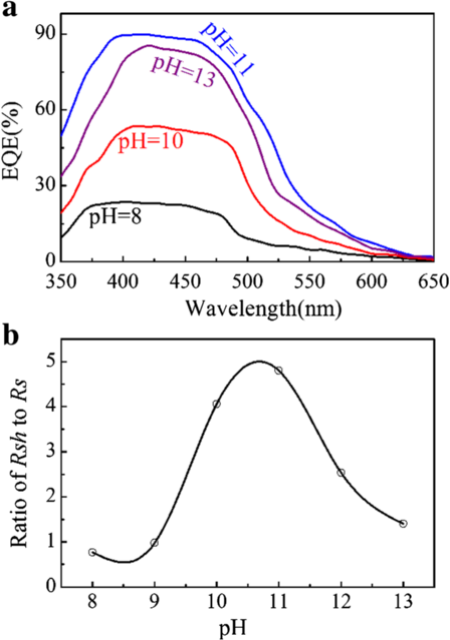
(Color online) **a** The EQE spectra of ZnO-NFs/Cu_2_O devices with Cu_2_O layers deposited at different pH values. **b** Ratio of *R*_sh_ to *R*_s_ for ZnO-NFs/Cu_2_O devices as a function of pH value

Subsequently, we come to clarify the origin of the *J*_sc_ dependence of the pH value. The measured EQE data for the cells prepared at different pH values for the Cu_2_O deposition are presented in Fig. 10a. A dramatic enhancement of the EQE in the visible range is observed upon the pH value increase from 8 to 11. The *J*_sc_ increment results from the improved absorption and efficient electron collection [31, 48]. The highest EQE at the pH value of ~11 suggests a good absorbance and charge transfer in this cell, thus indicating the best performance. Figure 10b show the plots for the ratio of shunt resistance (*R*_sh_) to series resistance (*R*_s_) for the cells fabricated at various pH values. The FF depends on the ratio of *R*_sh_ to *R*_s_ [48, 49]. One understands that the high FF value for the cell prepared at the pH value of ~11 is partially due to the large ratio of *R*_sh_ to *R*_s_. In short, this cell has the largest *J*_sc_ and FF, thus the best performances.

**Fig. 11:**
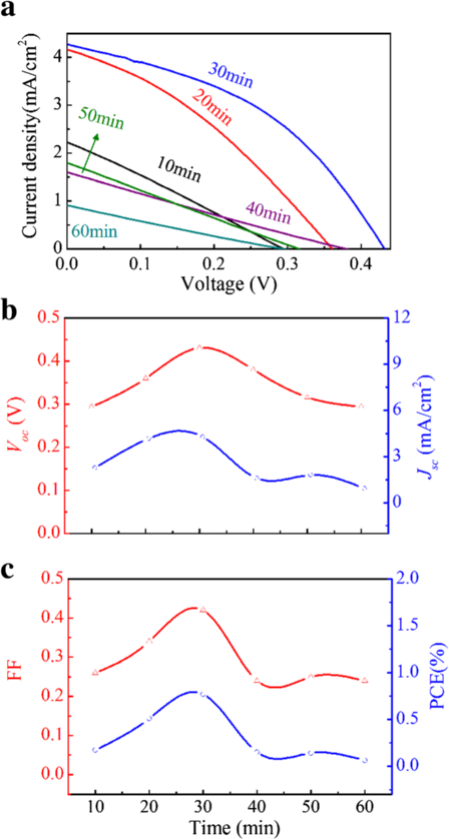
(Color online) **a** *J-V* curves, **b** *V*_oc_ and *J*_sc_, and **c** *FF* and PCE as a function of growth time for Cu_2_O layer in ZnO-NFs/Cu_2_O devices

Besides the substantial influence of the pH value, it is found that the Cu_2_O film thickness is also a critical parameter for the cell performances. It should be optimized to maintain a balance between the optical absorbance and charge transfer [25]. The effect of the optimized Cu_2_O thickness on performance of the cell is demonstrated by the different *J-V* curves obtained for different growth time for the Cu_2_O film, as shown in Fig. 11a. Fig. 11b, c shows the dependence of parameters *J*_sc_, *V*_oc_, FF, and PCE on the growth time. The PCE increases in the first 30 min and then decreases. The optimal growth time is 30 min, with the following obtained parameters: *V*_oc_~0.431 V, *J*_sc_~4.28 mA/cm^2^, FF~0.42, and PCE~0.77 %. The PCE is slightly higher than that of the cell that consists of no patterned AZO-ZnO nanorod arrays and electrodeposited Cu_2_O [39].

**Fig. 12:**
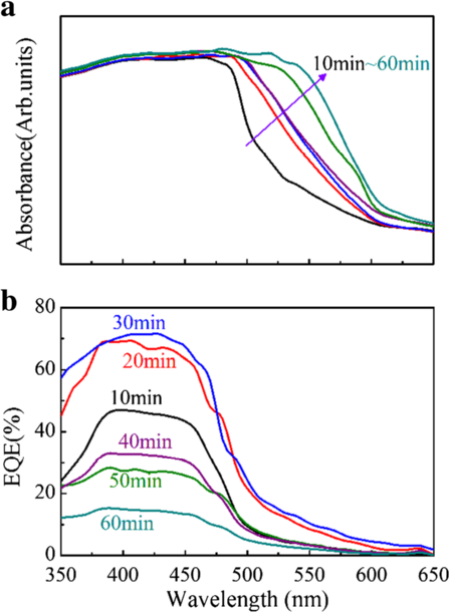
(Color online) **a** Absorbance and **b** external quantum efficiency (EQE) of ZnO-NFs/Cu_2_O devices with different growth time of Cu_2_O layer

It is believed that the optimized performance of a cell is attributed to two major factors: the enhanced absorption and balanced charge transfer. To clarify this issue, we present in Fig. 12 the absorption and EQE spectra of the cells fabricated at various growth time for Cu_2_O films. It is seen that the absorption increases with the growth time. The EQE intensity increases in the first 30 min and then decreases. As shown in Fig. 11, the *J*_sc_ reaches the maximum at the growth time of ~30 min. The *J*_sc_ increase in the first 30 min can be attributed to the enhanced *V*_bi_ and absorption [23]. Although the absorption can be further enhanced, a growth time longer than 30 min reduces the EQE and *J*_sc_. As shown in Fig. 2c, the Cu_2_O thickness is ~3.0 μm at the growth time of ~30 min, at which the *V*_bi_ is established [23]. An even longer growth time does not contribute to the *V*_bi_ on one hand, and the hole carriers have to travel through the thicker Cu_2_O, leading to reduced EQE and *J*_sc_.

**Fig. 13:**
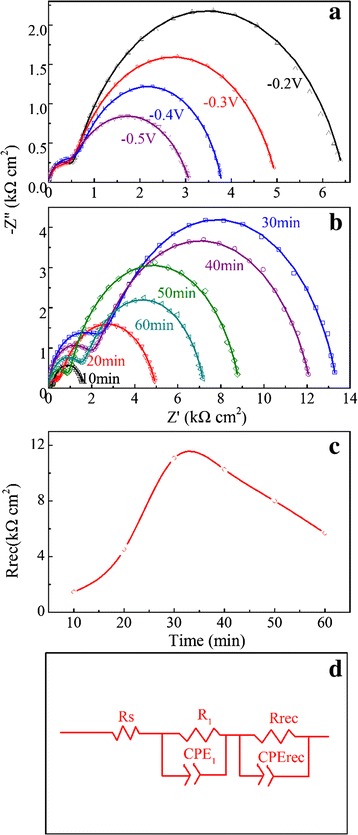
(Color online) **a** Example of Nyquist plots of ZnO-NFs/Cu_2_O device (20 min) as a function of bias voltages in the dark. **b** Nyquist plots of ZnO-NFs/Cu_2_O device with different growth time of Cu_2_O layer measured at the bias voltage of −0.3 V (close to *V*_oc_) in the dark. Data fits to impedance spectra are indicated by *solid lines*. **c** Recombination resistance of the ZnO-NFs/Cu_2_O devices obtained from **b** as a function of the growth time for Cu_2_O layer. **d** Simplified equivalent circuit employed to fit the Nyquist plots. *Solid lines* in **a** and **b** are the fittings of the experimental data using the model in **d**

Finally, we discuss the microscopic mechanisms for the thickness dependence of the cell performances, given that the Cu_2_O films are electrodeposited at the optimized pH value. The EIS data are used for evaluating the charge transfer and recombination at the junction interfaces. In our measurement, two semicircles are observed in the Nyquist plots for each cell. As an example, Fig. 13a shows the Nyquist plots of the EIS data as a function of bias voltages for cell where the growth time for the Cu_2_O film is 20 min. The plot shows two major features: one in the high-frequency range and the other in the low-frequency range. First, the arc in the high-frequency range is constant and independent of the applied bias, implying that it does not depend on the Fermi level position and can be ascribed to the parallel association of the geometrical capacitance constant phase element (CPE)_1_ and the charge transfer resistance *R*_1_ at the ZnO-ITO interfaces [23,40,50]. It is expected that this resistance varies very slowly with the bias voltage. Second, the large semicircle in the low-frequency range is ascribed to the parallel combination of the recombination resistance *R*_rec_ of electrons in the ZnO-NFs and holes in the Cu_2_O films, and the chemical capacitance *CPE*_rec_ from the *p–n* junction depletion region accounting for the inhomogeneities at the measured interfaces or from the Cu_2_O grain boundaries [40, 50, 51]. The size of the arc decreases as the applied bias voltage increases, as shown in Fig. 13a. This is due to the decrease in *R*_rec_ with the increase of electron density.

Subsequently, we compare the Nyquist plots for the cell at fixed bias voltage of −0.3 V (close to *V*_oc_) and the results are presented in Fig. 13b. The *solid lines* in Fig. 11a, b are the fits of experimental data using the model in the panel of Fig. 13d. For more accurate fitting, the CPE is used instead of the ideal capacitance *C* to account for spatial inhomogeneities that can be induced by defects and impurities at the interface. The measured Nyquist plots can indeed be fitted well. Figure 13c shows the fitted values of *R*_rec_ as a function of the growth time. By fitting the curve with the equivalent circuit, the parameters for ZnO-NFs/Cu_2_O junction are extracted. As shown in Fig. 13c, the value of *R*_rec_ increases in the first 30 min, and then decreases. It indicates that the recombination rate decreases upon the increasing growth time in the first stage because the recombination rate is inversely proportional to *R*_rec_ [52]. This will benefit for the charge transfer. When the growth time is longer than 30 min, the recombination rates increase. This result is consistent with the variation tendency of the *V*_oc_ and FF as a function of the growth time. These results confirm the fact that the growth time of ~30 min is the optimized one for the cells. Compared with the other cells, the better performance can be attributed to the effective charge transfer between the ZnO nanofibrous networks and Cu_2_O films and the higher absorption [37]. Figure 13d shows the equivalent circuit, where *R*_s_ is included due to contacts and wire.

**Fig. 14:**
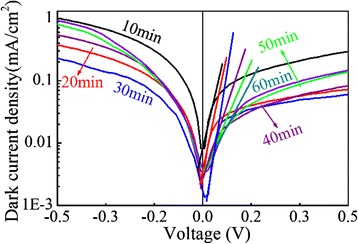
(Color online) Dark *J-V* curves of ZnO-NFs/Cu_2_O devices with different growth time for Cu_2_O layer

For further investigation, the dark *J-V* measurements are conducted for these samples too. Figure 14 shows the dark *J-V* curves of the cells with different growth time for the Cu_2_O films. The dark current primarily results from the recombination and electron leakage current at the interfaces of the heterojunctions [25]. For lattice-mismatched heterojunctions such as the ZnO-NFs/Cu_2_O cells, this dark current is expected to result from the recombination at the interfaces (holes from the Cu_2_O recombine with electrons from the ZnO) [20]. The dark current density decreases in the first 30 min but increases after 30 min in the forward bias. For the reverse bias, the leakage current decreases and then increases with the growth lasting, suggesting that more recombination events occur because of the increased Cu_2_O thickness. The ideality factor can be calculated from the slopes of the fitting straight lines in the linear regions at the low forward bias, which represents the interfacial recombination behavior. The larger slope corresponding to lower ideality factor represents a better diode performance [50]. As shown in Fig. 14, the slope increases with the deposition time from 10 to 30 min and then decreases. The ZnO-NFs/Cu_2_O device (growth time 30 min) shows the largest slope, thus the lowest ideality factor. It indicates the lowest interface states density in this device, which is consistent with the EIS results. And the lowest dark current density in the forward bias indicates less recombination sites and interfacial traps in the ZnO-NFs/Cu_2_O device. These results allow a conclusion that the ZnO-NFs/Cu_2_O device (growth time 30 min) not only exhibits efficient light harvesting but also low recombination rate and thus the best PCE.

## Conclusions

A series of inorganic solar cells based on electrospun ZnO nanofibrous networks and electrodeposited Cu_2_O films have been fabricated. We have demonstrated that controlling the pH value and growth time during the deposition of Cu_2_O layers can improve the performance of the ZnO-NFs/Cu_2_O cells significantly. The underlying mechanism is intrinsically related to the increased light absorption and balanced charge transfer at the ZnO-NFs/Cu_2_O heterojunctions. The measured PCE value of the as-prepared ZnO-NFs/Cu_2_O inorganic solar cell reaches up to 0.77 %.

## Additional file

Additional file 1
**Figure S1**. (Color online) The (111)/(200) peak intensity ratio as a function of pH value. The peak intensity data are taken from the XRD measurement in Fig. 4. **Figure S2**. (Color online) J-V curves of the ZnO film/Cu_2_O and ZnO-NFs/Cu_2_O devices fabricated at pH = 11. **Figure S3.** (Color online) The M-S plots of ZnO-NFs. **Figure S4**. (Color online) The Nyquist plots of the ZnO-NFs/Cu_2_O devices with various pH values for Cu_2_O deposition, measured at the bias voltage of −0.3 V (close to *V*_oc_) in the dark. The solid lines are the fittings of experimental data using the model in Fig. 11d.
