# Supplementary material for: Inorganic Solar Cells Based on Electrospun ZnO Nanofibrous Networks and Electrodeposited Cu2O
Source: Nanoscale Res Lett. 2015 Dec 1;10:465. doi: 10.1186/s11671-015-1169-8 (PMC4666848; doi:10.1186/s11671-015-1169-8)
Supplement: Additional file 1 — Figure S1. (Color online) The (111)/(200) peak intensity ratio as a function of pH value. The peak intensity data are taken from the XRD measurement in Fig. 4. Figure S2. (Color online) J-V curves of the ZnO film/Cu2O and ZnO-NFs/Cu2O devices fabricated at pH = 11. Figure S3. (Color online) The M-S plots of ZnO-NFs. Figure S4. (Color online) The Nyquist plots of the ZnO-NFs/Cu2O devices with various pH values for Cu2O deposition, measured at the bias voltage of −0.3 V (close to V oc) in the dark. The solid lines are the fittings of experimental data using the model in Fig. 11d. [file 11671_2015_1169_MOESM1_ESM.docx]

**Supporting Information**

**Inorganic solar cells based on electrospun ZnO nanofibrous networks and electrodeposited Cu_2_O**

Luming Zhang^a^, Huaquan Sun^a^, Lai Xie^a^, Jinnan Lu^a^, Luyong Zhang^a^, Sujuan Wu^a*^, Xingsen Gao^a^, Xubing Lu^a^, Jinhua Li^b^ and Jun-Ming Liu^a,c^[[1]](#footnote-1)^*^

^a^*Institute for Advanced Materials and Guangdong Provincial Key Laboratory of Quantum Engineering and Quantum Materials, South China Normal University, Guangzhou 510006, China*

^b^*Faculty of Materials Science and Engineering, Hubei University, Wuhan, 430062,*

*China*

^c^*Laboratory of Solid State Microstructures, Nanjing University, Nanjing 210093, China*

E-mail: sujwu@scnu.edu.cn , [liujm@nju.edu.cn](mailto:liujm@nju.edu.cn)





**Figure S1.** (Color online) The (111)/(200) peak intensity ratio as a function of pH value. The peak intensity data are taken from the XRD measurement in Figure 4.





**Figure S2.** (Color online) J-V curves of the ZnO flim/Cu_2_O and ZnO-NFs/Cu_2_O devices fabricated at pH=11.


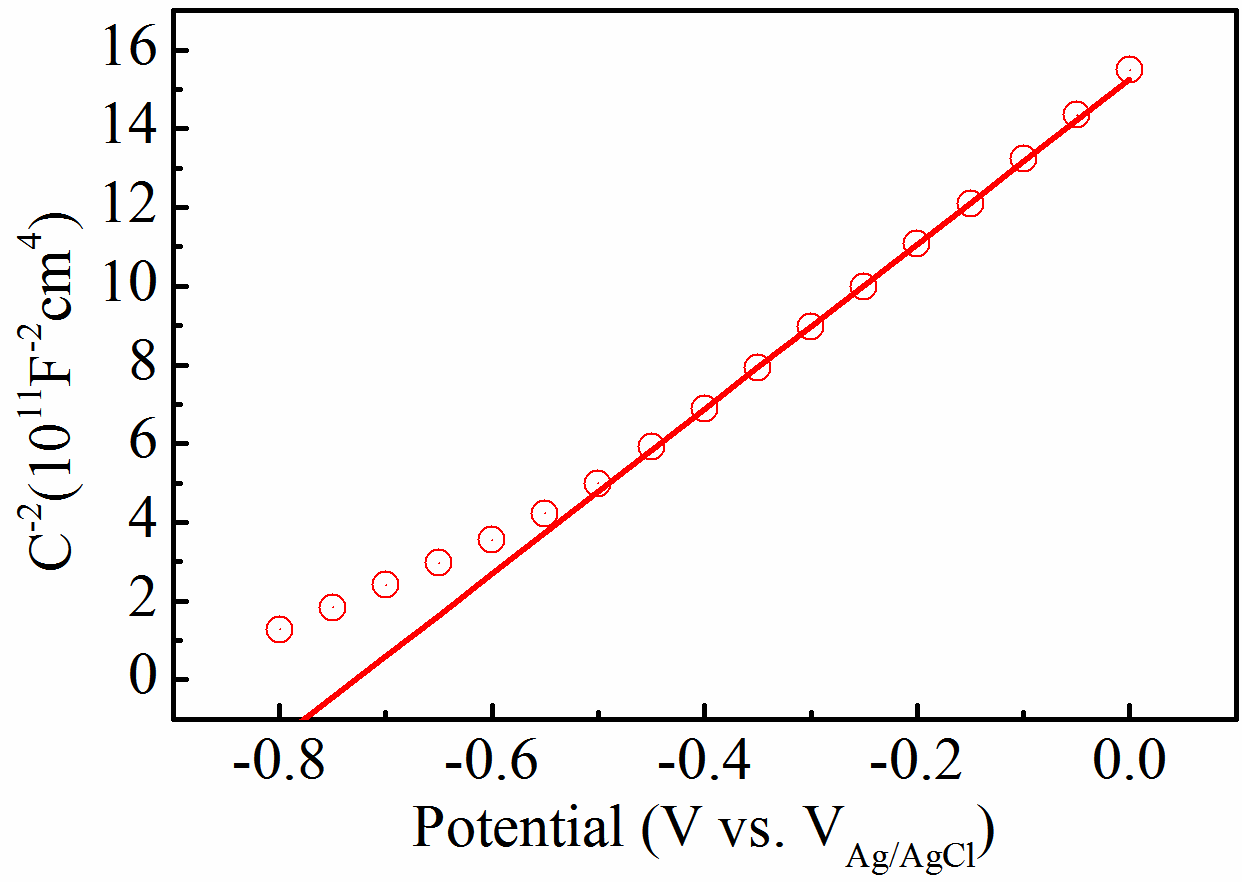


**Figure S3.** (Color online) The M-S plots of ZnO-NFs.


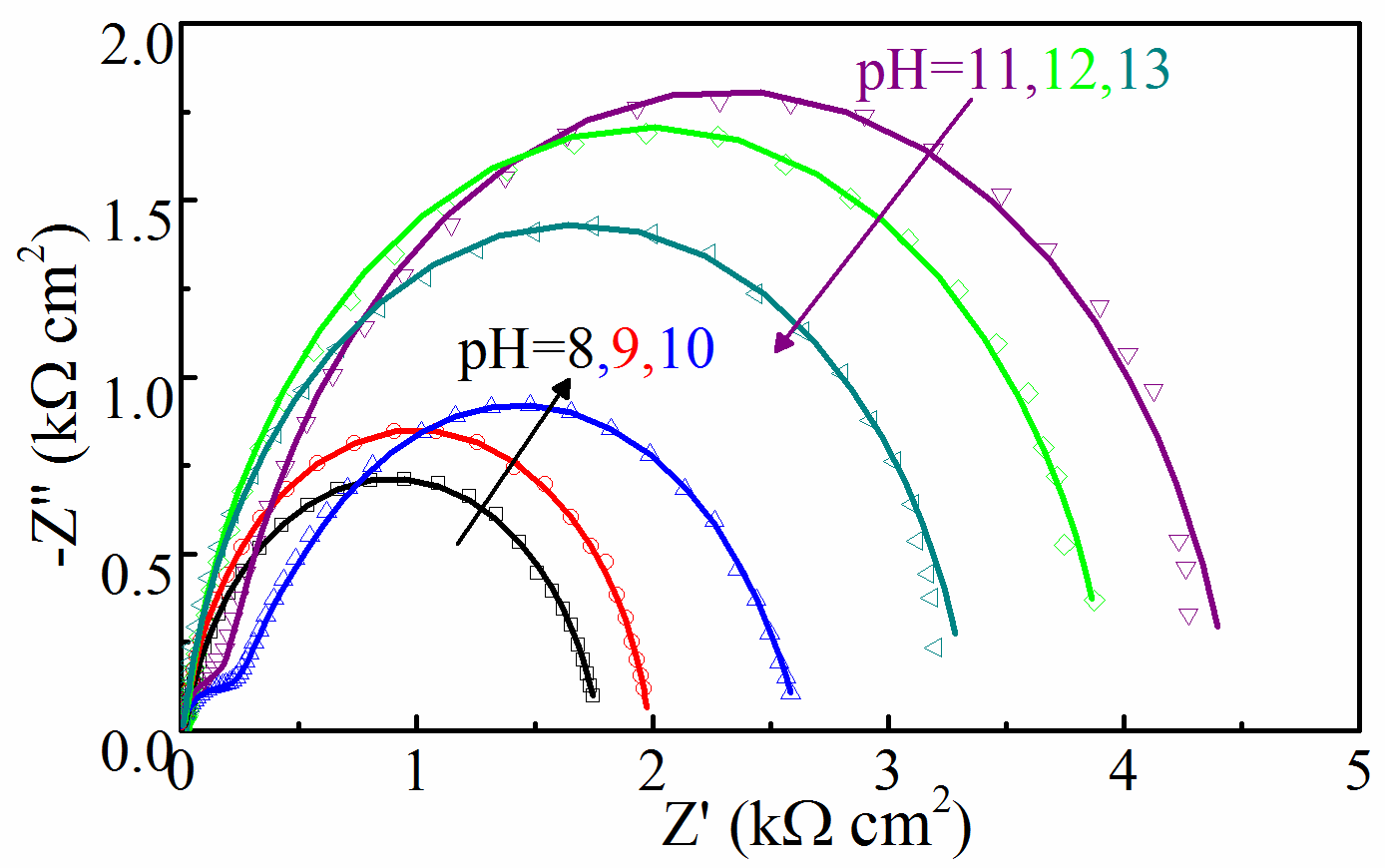


**Figure S4.** (Color online) The Nyquist plots of the ZnO-NFs/Cu_2_O devices with various pH values for Cu_2_O deposition, measured at the bias voltage of -0.3V (close to V_oc_) in the dark. The solid lines are the fittings of experimental data using the model in Figure 11(d).

1. * Corresponding authors, E-mail: [sujwu@scnu.edu.cn](mailto:sujwu@scnu.edu.cn) and E-mail: [liujm@nju.edu.cn](mailto:liujm@nju.edu.cn) [↑](#footnote-ref-1)
